# Opposing Roles of Leptin and Ghrelin in the Equine Corpus Luteum Regulation: An *In Vitro* Study

**DOI:** 10.1155/2014/682193

**Published:** 2014-07-14

**Authors:** António Galvão, Angela Tramontano, Maria Rosa Rebordão, Ana Amaral, Pedro Pinto Bravo, Anna Szóstek, Dariusz Skarzynski, Antonio Mollo, Graça Ferreira-Dias

**Affiliations:** ^1^C.I.I.S.A., Faculty of Veterinary Medicine, University of Lisbon, Portugal; ^2^Institute of Animal Reproduction and Food Research of PAS, Olsztyn, Poland; ^3^Department of Animal Medicine, Production and Health (MAPS), Faculty of Veterinary Medicine, University of Padua, Viale dell'Università 16, 35020 Legnaro, Padua, Italy

## Abstract

Metabolic hormones have been associated with reproductive function modulation. Thus, the aim of this study was: (i) to characterize the immunolocalization, mRNA and protein levels of leptin (LEP), Ghrelin (GHR) and respective receptors LEPR and Ghr-R1A, throughout luteal phase; and (ii) to evaluate the role of LEP and GHR on progesterone (P_4_), prostaglandin (PG) E_2_ and PGF_2*α*_, nitric oxide (nitrite), tumor necrosis factor-*α* (TNF); macrophage migration inhibitory factor (MIF) secretion, and on angiogenic activity (BAEC proliferation), in equine corpus luteum (CL) from early and mid-luteal stages. LEPR expression was decreased in late CL, while GHR/Ghr-R1A system was increased in the same stage. Regarding secretory activity, GHR decreased P_4_ in early CL, but increased PGF_2*α*_, nitrite and TNF in mid CL. Conversely, LEP increased P_4_, PGE_2_, angiogenic activity, MIF, TNF and nitrite during early CL, in a dose-dependent manner. The *in vitro* effect of LEP on secretory activity was reverted by GHR, when both factors acted together. The present results evidence the presence of LEP and GHR systems in the equine CL. Moreover, we suggest that LEP and GHR play opposing roles in equine CL regulation, with LEP supporting luteal establishment and GHR promoting luteal regression. Finally, a dose-dependent luteotrophic effect of LEP was demonstrated.

## 1. Introduction 

A plethora of factors control ovarian function, evidencing the biological complexity of its regulation [1]. Luteal function and fate are controlled by steroid hormones, prostaglandins, nitric oxide, angiogenic and antiangiogenic factors, and numerous cytokines, such as tumor necrosis factor *α* (TNF), interferon-*γ*, or Fas/Fas ligand system [2–5]. Additionally, other hormones and metabolic factors like leptin (LEP) or ghrelin (GHR), which are involved in energetic balance regulation and metabolism, also modulate gonadal axis function [6]. Particularly, both LEP and GHR may operate as endo- and paracrine mediators connecting energy balance and reproductive tract [7]. Recent reports demonstrate the expression of LEP and GHR and their receptors in different reproductive organs, such as ovary [8], endometrium [9], embryo, or placenta [10]. In the mare,* in vivo* LEP effect on seasonal ovarian cyclicity and fertility was previously addressed [11, 12].

Ghrelin, the endogenous ligand of growth hormone (GH) secretagogue receptor type 1a designated as the GHR receptor (Ghr-R1A), is a pleiotropic factor secreted mainly by the oxyntic glands in the stomach [13]. This hormone is involved in a large array of endocrine and nonendocrine functions, like cell proliferation or apoptosis regulation [14], energy homeostasis, and orexigenic effect [15]. Regarding the reproductive function, GHR was shown to inhibit both* in vivo* and* in vitro* LH secretions in rats under negative energetic balance (fasting or anorexia) and to decrease* in vitro* LH responsiveness to GnRH [16]. Expression of GHR has been lately demonstrated in several tissues and cell types, such as placenta, testis, and ovary of human, rat, pig and sheep, and chicken [17–19].

Leptin is an adipocyte-derived hormone (adipokine) under the control of the obesity (*ob*) gene [20]. Leptin signaling is accomplished via membrane receptors belonging to the Class I cytokine family [21]. The primary biological role can be attributed to the long form (LEPR), containing a complete intracellular domain, capable of activating the JAK-STAT signaling pathway. This domain is responsible for the majority of the biological effects of LEP [22]. The expression of ovarian LEPR and its involvement on ovarian function were demonstrated in human [23], mouse [24], rat [25], porcine [26], and bovine [27].

Although these metabolic factors have been shown to affect ovarian function in different species, their role in equine corpus luteum (CL) is still unknown. Thus, we hypothesize that the locally produced hormones LEP and GHR modulate equine CL function, regulating its secretory activity and angiogenic function throughout the luteal phase. The goals of the study are (i) to characterize cellular immunolocalization (immunohistochemistry) and expression profile (mRNA and protein levels) of LEP, LEPR, GHR, and Ghr-R1A throughout the luteal phase; (ii) to evaluate the role of the hormones LEP and GHR in secretory activity (progesterone (P_4_)_,_ prostaglandin (PG) E_2_ and PGF_2*α*_, nitric oxide (nitrite), tumor necrosis factor *α* (TNF), and macrophage migration inhibitory factor (MIF, a proangiogenic factor)) and angiogenic activity (using bovine aortic endothelial cells proliferation assay) during CL establishment and maintenance.

## 2. Materials and Methods

### 2.1. Animals and CL Tissue Collection

Corpora lutea from cyclic mares were collected* post-mortem* from April until the end of July at a local abattoir from randomly selected cyclic Lusitano mares. The mares were healthy as stated by official governmental veterinary inspection. Material collection followed the previously described [4, 5]. The mares were euthanized after stunning according to the European Legislation concerning welfare aspects of animal stunning and killing methods (EFSA, AHAW/04-027) and the Portuguese legislation (DL 98/96, Art. 1°) and as approved by the Faculty of Veterinary Medicine Ethics Committee.

After euthanasia of the mares, internal reproductive organs were collected. The ovaries were isolated and opened up and luteal structures were classified as follows [4]: early luteal phase CL (presence of corpus hemorrhagicum, P_4_ > 1 ng/mL, early CL; *n* = 11), midluteal phase CL (CL associated with follicles 15 to 20 mm in diameter and P_4_ > 6 ng/mL, mid-CL; *n* = 11), or late luteal phase CL (CL associated with a preovulatory follicle 30–35 mm in diameter and P_4_ between 1 and 2 ng/mL, late CL; *n* = 6). For tissue culture, corpora lutea were collected within 5 min of death, placed in sterile culture medium M199 (M2154; Sigma-Aldrich, St. Louis, MO, USA) supplemented with gentamicin (20 *μ*g/mL; G1272, Sigma), amphotericin (250 *μ*g/mL; A2942, Sigma), and 0.1% (w/v) bovine serum albumin (BSA; Sigma, A9056), kept on ice, and transported to the laboratory. Additionally, the CL tissue was excised, rinsed with cold sterile RNAse-free saline solution, divided into three groups concerning the experimental analysis, and placed in (i) 4% buffered formaldehyde for immunolocalization staining (IHC); (ii) RNA later (Invitrogen, AM7021) for real-time PCR, or (iii) liquid nitrogen for Western blot.

### 2.2. Luteal Tissue Explant Culture

Luteal tissue isolation followed the methodology described before [28]. Briefly, CL explants were minced into small pieces and 40 mg of tissue was washed three times with a sterile phosphate buffer solution (PBS) containing gentamicin (50 *μ*g/*μ*L) and placed into culture tubes containing 1 mL Dulbecco's modified Eagle's medium (DMEM) and Ham's F-12 medium (D/F medium; 1 : 1 [v/v], D-8900, Sigma) and supplemented with 0.1% BSA and 1% antibiotic and antimycotic solution (Sigma, A5955). Tissue explants were preincubated on a shaker at 37.0°C with 5% CO_2_ in air for 1.5 h and then medium was replaced with fresh DMEM supplemented with 0.1% BSA and antibiotics and antimycotic.

### 2.3. Experimental Procedures

#### 2.3.1. Experiment 1: Characterization of LEP, LEPR, GHR, and Ghr-R1A Expression and Cellular Localization in the Equine CL

Samples from early (*n* = 6), mid- (*n* = 6), and late (*n* = 6) CL were used. Immunohistochemistry (IHC), real-time PCR, and Western blot were performed in order to identify the cellular immunolocalization of LEP and GHR and their receptors, as well as mRNA transcription and protein expression.

*Immunohistochemistry*. The histological sections were immunostained for localization of LEP, GHR, LEPR, and GHR-R1A in CL, according to Galvao et al. [4]. Briefly, the sections were incubated with primary antibodies against LEP (rabbit polyclonal diluted 1 : 100, ab16227, Abcam, Cambridge, UK), GHR (mouse monoclonal diluted 1 : 500, ab57222, Abcam), LEPR (rabbit polyclonal diluted 1 : 100, ab104403, Abcam), and Ghr-R1A (rabbit polyclonal diluted 1 : 200, ab85104, Abcam). Immunohistochemistry staining was assessed as a characteristic brown staining, with a light microscope (Olympus BX51, Tokyo, Japan). Tissue areas were photographed (DP11 Olympus, Tokyo, Japan).

*Total RNA Isolation and cDNA Synthesis*. Total RNA was extracted from luteal tissue as described before [4] using Qiagen's kit for total RNA extraction and purification (28704, Qiagen, Hilden, Germany) and DNA digested (RNase-free DNase Set, 50979254, Qiagen) according to the manufacturer's instructions. Both RNA concentration and quality were determined spectrophotometrically and by agarose gel electrophoresis. The ratio of absorbance at 260 nm and 280 nm (A_260/280_) was approximately 2. The RNA (1 *μ*g) was reversed transcribed into cDNA using a ThermoScript RT-PCR System (Qiagen) according to the manufacturer's instructions. The cDNA was stored at −20°C until real-time PCR was carried out.

*Real-Time PCR*. Real-time PCR was performed as before [4, 5], with ABI Prism 7300 sequence detection system using SYBR green PCR master mix (Applied Biosystems, Foster City, CA, USA). Based on gene sequences in GenBank (NCBI), the primers for* LEP*,* GHR*,* LEPR,* and* GHR-R1A* were designed using Primer Express 3.0 software (Applied Biosystems). All primers were synthesized by Genomed (Warsaw, Poland). Primer sequences, expected PCR products length, and GenBank accession numbers of* LEP*,* GHR*,* LEPR,* and* Ghr-R1A* are reported in Table 1. Total reaction volume was 20 *μ*L containing 5 *μ*L water, 1 *μ*L cDNA, 2 *μ*L each forward and reverse primers (80 nM), and 10 *μ*L SYBR green PCR master mix. Real-time PCR was carried out as follows: initial denaturation (10 min at 95°C), followed by 40 cycles of denaturation (15 s at 95°C) and annealing (1 min at 60°C). After each PCR reaction, melting curves were obtained by stepwise increases in temperature from 60 to 95°C to ensure single product amplification. The specificity of product was also confirmed by electrophoresis on 2% agarose gel. *β*2-Microglobulin (*β*2MG) was used as housekeeping gene. The data were analyzed using the method described by [29].

*Western Blot.* For immunoblotting, protein fractions were obtained from total tissue protein, following the methodology previously described [4, 5]. Tissues were minced and placed in ice-cold RIPA buffer (50 mM Tris-HCl, pH 7.4, 50 mM EDTA, 150 mM NaCl, and 1% Triton X-100) with protease inhibitor (11697498 001, Roche Diagnostics Poland, Warsaw, Poland) and homogenized on ice. After protein extraction and determination of protein concentrations using Bicinchoninic Acid Protein Assay Kit (23225, Thermo Scientific, Rockford, IL, USA), a total of 60 *μ*g protein was loaded onto an acrylamide gel (161-0155, Bio-Rad). Then, proteins were transferred to nitrocellulose membranes (1620116, Bio-Rad). Protein levels were evaluated with the antibodies used in immunohistochemistry diluted 1 : 400 for LEP, 1 : 200 for GHR, 1 : 400 for LEPR, and 1 : 200 for Ghr-R1A and incubated overnight in 4°C. To normalize the protein loading, a mouse monoclonal mouse antibody against *β* actin (A5441, Sigma) was used at a dilution 1 : 10000. Then, the proteins were detected by incubating the membrane with secondary polyclonal anti-rabbit alkaline phosphatase-conjugated antibody (1 : 30000 for LEP, LEPR, and Ghr-R1A, A3812, Sigma) or secondary polyclonal anti-mouse alkaline phosphatase-conjugated antibodies (dilution 1 : 30000 for GHR and *β* actin; A3562, Sigma) for 1.5 h at room temperature. After washing in TBS-T buffer, immune complexes were visualized using the alkaline phosphatase visualization procedure. For densitometric analyses, the blots were scanned and specific bands were quantified using Kodak 1D Image Analysis Software (Eastman Kodak). Finally, band density for each of the target protein was normalized against *β* actin.

#### 2.3.2. Experiment 2: The Influence of Leptin and Ghrelin on Secretory Function of Equine CL* In Vitro*

Luteal samples were obtained from mare at early (*n* = 5) and mid- (*n* = 5) luteal phase. The tissue culture was prepared as described above. Explants were exposed to (i) no factor (negative control); (ii) GHR (G3902, Sigma; 50 ng/mL); (iii) LEP (L4146, Sigma; 5 ng/mL); (iv) LEP (200 ng/mL); (v) GHR+LEP 5 ng/mL; or (vi) GHR+LEP 200 ng/mL for 24 h. The most effective dose and the optimal treatment time for LEP and GHR action were established in a preliminary study regarding P_4_ secretion. For LEP, the physiologic doses of 1 and 5 ng/mL and supraphysiologic doses of 200 and 500 ng/mL were tested; for GHR, the doses of 5, 50, and 100 were tested (data not shown); and luteinizing hormone (LH, 10 ng/mL) (30) was used as a positive control. After incubation, conditioned culture medium was collected and kept frozen at −20°C until P_4_, PG, TNF, MIF, and nitrite determination. In order to normalize results, concentration of hormones was assessed per 1 g of viable tissue, measured by alamarBlue reagent method (AbD Serotec, Oxford, UK; BUF012A), following the manufacturer's guide and as briefly described below.

#### 2.3.3. Experiment 3: The Influence of Leptin and Ghrelin Treatment on Angiogenic Activity

The effect of different treatments on angiogenic activity in early CL and mid-CL tissue-conditioned culture media (Luteal Conditioned Media, LCM) was indirectly assessed after viability evaluation of bovine aortic endothelial cells (BAEC, kindly donated by Dr. D. A. Redmer, Department of Animal and Range Sciences, North Dakota State University, Fargo, ND, USA), using alamarBlue reagent (alamarBlue reagent, Serotec, Oxford, UK). Protocol optimization for BAEC was described elsewhere [3, 30]. Briefly, BAEC (2 × 10^4^ cells/mL) was incubated in 24-well plates at 37°C in a humidified atmosphere (5% CO_2_ and 95% air) for 14 h until the cells adhered to the wells. Thereafter, media were changed to treatment-conditioning medium (TCM), which consisted of 30% LCM (Experiment 1) and 70% fresh serum free D/F medium. In negative controls, TCM was replaced by culture medium alone, that is, without luteal tissue, containing the same treatment factors used for experimental treatments. Samples were run in triplicate and incubated for 48 h. The TCM was then removed and fresh phenol red-free D/F medium containing 10% alamarBlue was added. The plates were incubated for the next 5 h and absorbance (abs) read at 570 and 600 nm (SpectrMax 340 PC; Molecular Devices; Biocitek SA, Lisbon, Portugal). The optimal incubation time for BAEC with conditioned media from equine luteal tissue cultures was 5 h, since at this time a linear correlation between the percentage reduction of the indicator and cell density was the highest (*R* ^2^ = 0.9507), according to the manufacturer's instructions [3, 30]. The percentage of viable BAEC after incubation with TCM was evaluated by comparing the percentage reduction of the media indicator with that produced by the negative controls (without luteal tissue). Cell viability or mitogenesis in response to negative controls was considered to be 100%. Reduction or oxidation of the media indicator was evaluated by cellular incorporation of the fluorimetric/colourimetric growth indicator. alamarBlue percentage reduction using abs was determined according to the technical datasheet.

### 2.4. Analytical Methods

#### 2.4.1. Prostaglandins Determination

The concentration of PGE_2_ in the conditioned medium was determined using a prostaglandin E_2_ EIA kit (Cayman Chemical Company, Ann Arbor, USA) according to the manufacturer's instructions. The concentration of PGF_2*α*_ was determined using the direct enzyme immunoassay (EIA) method as previously described by Uenoyama et al. [31] with modifications. The standard curve for PGE_2_ ranged from 16.5 ng/mL to 1000 ng/mL. The intra- and interassay coefficients of variation (CV) were 5.9% and 7.6%, respectively. The standard curve for PGF_2*α*_ ranged from 0.19 ng/mL to 50 ng/mL and CV were 5.5% and 8.6%, respectively.

#### 2.4.2. Progesterone Determination

Concentration of P_4_ in blood plasma and luteal explant conditioned media was determined by EIA as described previously [32]. The standard curve for P_4_ ranged from 0.0925 ng/mL to 25 ng/mL and intra- and interassay CV were 4.8% and 6.7%, respectively.

#### 2.4.3. MIF Determination

The concentration of MIF in the conditioned medium was determined using MIF DuoSet ELISA (DY289, R&D Systems, Abingdon, UK) according to the manufacturer's instructions. The MIF standard curve ranged from 16.5 to 2000 pg/mL. The intra- and interassay CV were 6.7% and 7.5%, respectively.

#### 2.4.4. TNF Determination

The concentration of TNF in the conditioned medium was performed as described before [33] using TNF*α* DuoSet ELISA (DY2279, R&D Systems) according to the manufacturer's instructions. The TNF standard curve ranged from 16.5 to 2000 pg/mL. The intra- and interassay CV were 6.1% and 5.5%, respectively.

#### 2.4.5. Nitrite Determination

The concentration of nitrite in the conditioned medium was determined using Griess Reagent System (Promega, Madison, USA; number G2930), according to the manufacturer's instructions. The amount of nitrite produced was determined spectrophotometrically as formed nitrite, and its content was calculated on the basis of a standard curve constructed using NaNO_2_ (0–100 M) as described before [34].

#### 2.4.6. Statistical Analysis

The data are shown as the mean ± SEM of values obtained in separate experiments, each performed in triplicate. The statistical analysis of data from Experiment 1 was performed using nonparametric one-way ANOVA* Kruskal*-*Wallis* followed by* Dunn's test* (GraphPad Software version 5, San Diego, USA). The statistical analysis of data from Experiment 2 was performed using parametric one-way ANOVA followed by* Newman-Keuls* comparison test. The results were considered significantly different when *P* < 0.05.

## 3. Results

### 3.1. Experiment 1: Characterization of LEP, LEPR, GHR, and Ghr-R1A Expression and Cellular Localization in the Equine CL

**Figure 1:**
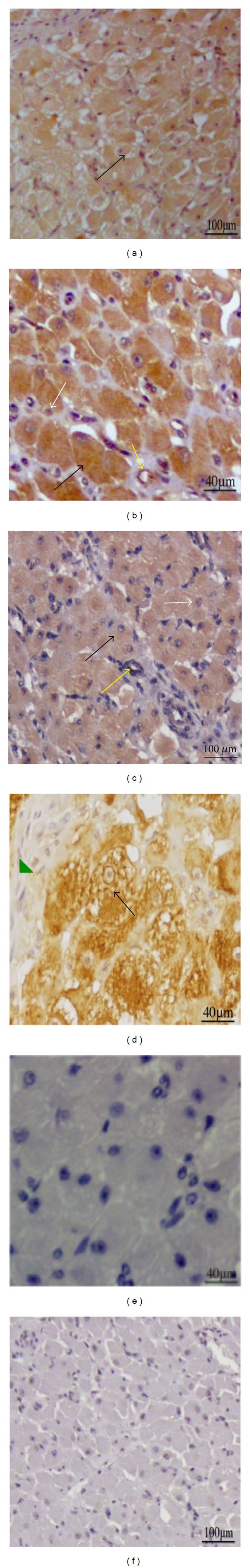
Representative images of equine CL immunostained for the presence of LEP in early CL (a), LEPR in mid-CL (b), GHR in mid-CL (c), Ghr-R1A in late CL ((d) characteristic increased number of vacuole in LLC and connective tissue for late CL-green triangle). Negative control with substitution of primary antibody by rabbit IgG (e) and by PBS (f). Black arrow indicates LLC, white arrow indicates SLC, and yellow arrow indicates endothelial cells. Since all cytokines/receptors stained equally throughout the estrous cycle, pictures from each luteal phase were randomly assigned.

Immunohistochemistry depicted the presence of the ligands LEP and GHR, together with their respective receptors, LEPR and Ghr-R1A, in equine luteal cells. No staining was present in negative controls (Figures 1(e) and 1(f)). Ligands LEP and GHR and receptors LEPR and Ghr-R1A were immunolocalized in small luteal cells (SLC) (Figures 1(b) and 1(c), white arrow), large luteal cells (LLC) (Figures 1(a), 1(b), 1(c), and 1(d), black arrow), and endothelial cells (Figures 1(b) and 1(c), yellow arrow). Once no differences were observed between factors and stages of the luteal phase, figures were randomly assembled.

**Figure 2:**
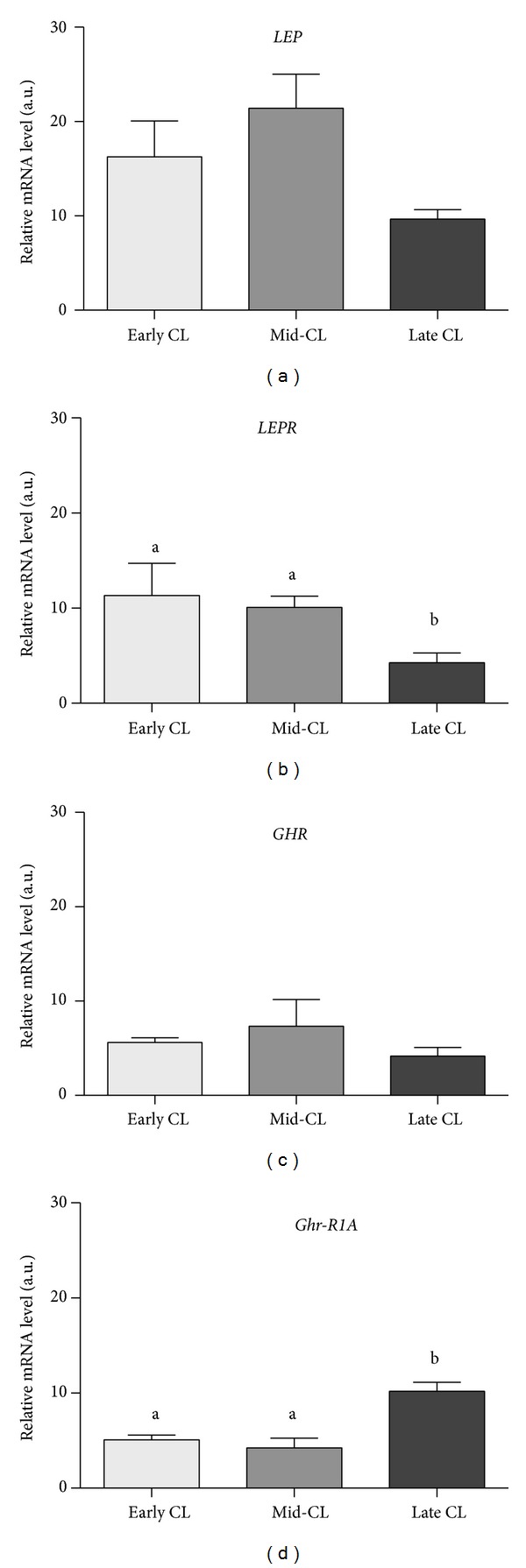
Relative quantification of* LEP* (a),* LEPR* (b),* GHR* (c), and* Ghr-R1A* and (d) mRNA level by real-time PCR. Transcription normalized with the housekeeping gene* B2MG*. Bars represent mean ± SEM. Different letters indicate significant differences (*P < 0.05; ***P < 0.001).

Regarding mRNA transcription, no significant changes were found in ligands* LEP* and* GHR* (Figures 2(a) and 2(c)) throughout the luteal phase, while* LEPR* mRNA level was decreased in late CL (*P* < 0.05, Figure 2(b)) and* Ghr-R1A* was increased (*P* < 0.05, Figure 2(b)) in the same stage of the cycle.

**Figure 3:**
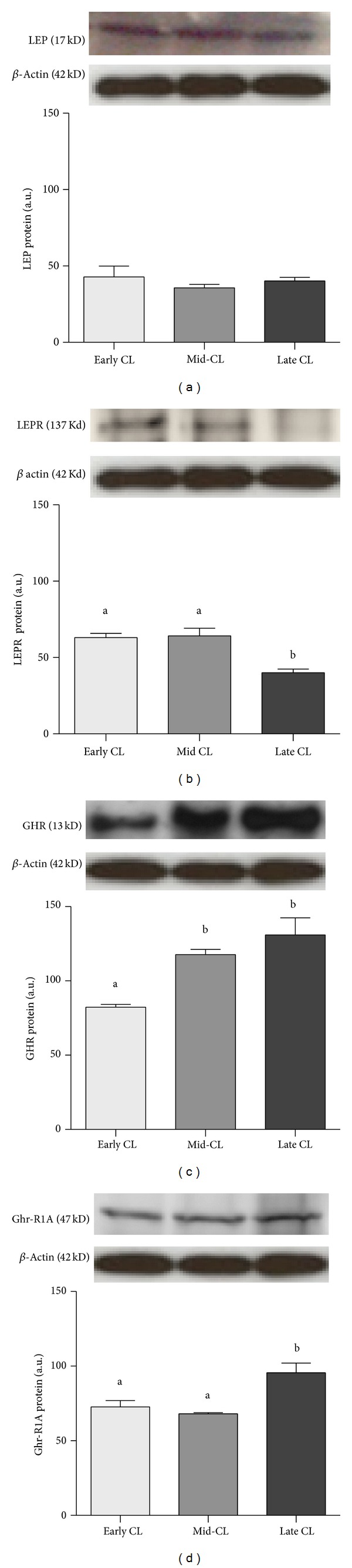
Protein expression of LEP (a), LEPR (b), GHR (c), and Ghr-R1A (d). Upper panels depict representative Western blotting (*n* = 4). Data normalized against *β* actin density values. Bars represent mean ± SEM. Different letters indicate significant differences (*P < 0.05; **P < 0.01).

Protein expression analysis by western blot showed no changes in band intensity for LEP (Figure 3(a)), but a decrease in the expression level of LEPR from mid- to late CL was present (*P* < 0.05, Figure 3(b)). With respect to GHR system, GHR presented a raise in protein level from early CL to mid-CL, being highly expressed still in late CL (*P* < 0.05, Figure 3(c)). The expression of Ghr-R1A increased from mid- to late CL (*P* < 0.05, Figure 3(d)).

### 3.2. Experiment 2: Effect of LEP and GHR on Progesterone and Prostaglandins Secretion

**Figure 4:**
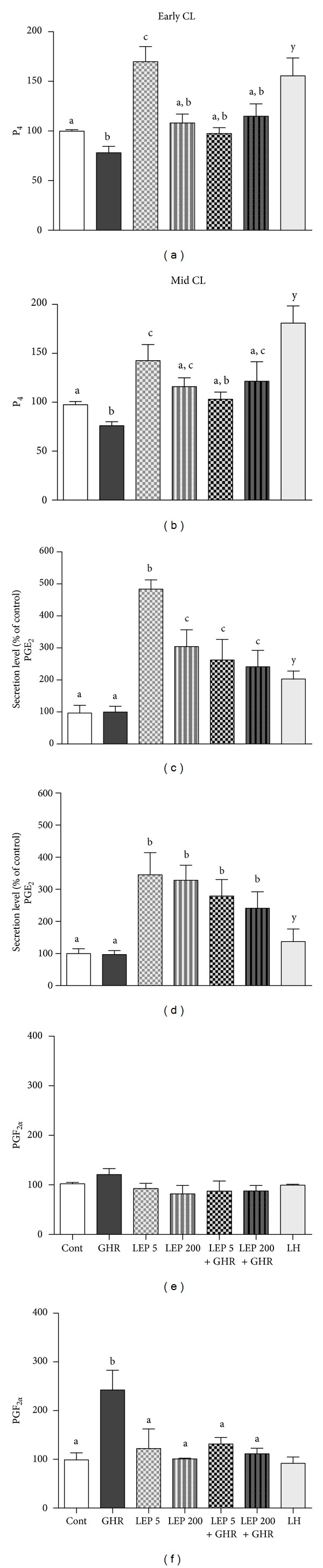
Early CL and mid-CL explants* in vitro* production of P_4_ ((a) and (b)); PGE_2_ ((c) and (d)); and PGF_2*α*_ ((e) and (f)), after 24 h treatment with no exogenous factor = Control or with GHR (50 ng/mL), LEP (5 ng/mL), LEP (200 ng/mL), LEP (5 ng/mL)+GHR (50 ng/mL), LEP (200 ng/mL)+GHR (50 ng/mL), or LH (positive control; 10 ng/mL). Bars represent mean ± SEM. Different letters indicate significant differences (*P < 0.05; **P < 0.01; ***P < 0.001). Correspondent value of control for hormone production mean ± SEM: early CL P_4_ (1.05 ± 0.031 ng/mg); mid-CL P_4_ (6.305 ± 0.30 ng/mg) and for PGE_2_ (0.23 ± 0.05 ng/mg) and PGF_2*α*_ (0.147 ± 0.015 ng/mg).

The tissue culture system previously validated [28] allowed for the study of LEP and GHR role on CL secretory activity. Assessment of tissue viability with alamarBlue (data not shown) together with the analysis of LH treatment responsiveness (positive control) allowed for the determination tissue viability in Experiment 2. Treatment with GHR decreased P_4_ secretion together in early CL (a; b = *P* < 0.05, Figure 4(a)) and mid-CL (a, b = *P* < 0.05, Figure 4(b)). Conversely, treatment with LEP 5 ng/mL increased P_4_ production by luteal tissue in early CL (a; c = *P* < 0.05, Figure 4(a)) and mid-CL (a; c = *P* < 0.05, Figure 4(b)). Leptin 200 ng/mL caused no significant changes in P_4_ level in culture media (Figures 4(a) and 4(b)). When LEP 5 ng/mL and GHR were used in association, LEP stimulatory effect was significantly reverted (c; a, b = *P* < 0.05, Figures 4(a) and 4(b)) and no changes in P_4_ production were seen, comparing to the control. No changes were observed in P_4_ after GHR+LEP 200 ng/mL (Figures 4(a) and 4(b)). Considering PGE_2_ regulation, while GHR had no effect on both early CL and mid-CL stages (Figures 4(c) and 4(d)), LEP at 5 and 200 ng/mL concentration, alone or in association with GHR, consistently increased PGE_2_ secretion (*P* < 0.05, Figures 4(c) and 4(d)). The stimulatory effect of LEP on PGE_2_ in early CL depended on treatment dose. Treatment LEP 5 ng/mL caused a more significant increase of PGE_2_ (a; b = *P* < 0.001, Figure 4(c)) than LEP 200 ng/mL (a; c = *P* < 0.05, Figure 4(c)). The PGF_2*α*_ secretion was changed exclusively in mid-CL, with GHR stimulating its output (a; b = *P* < 0.01, Figure 4(f)). The positive control LH increased P_4_ and PGE_2_ in both early CL and mid-CL in a significant manner (a; y = *P* < 0.05, Figure 4).

### 3.3. Experiment 3: The Influence of Leptin and Ghrelin Treatment on Angiogenic Activity, TNF, and Nitrite Secretion

**Figure 5:**
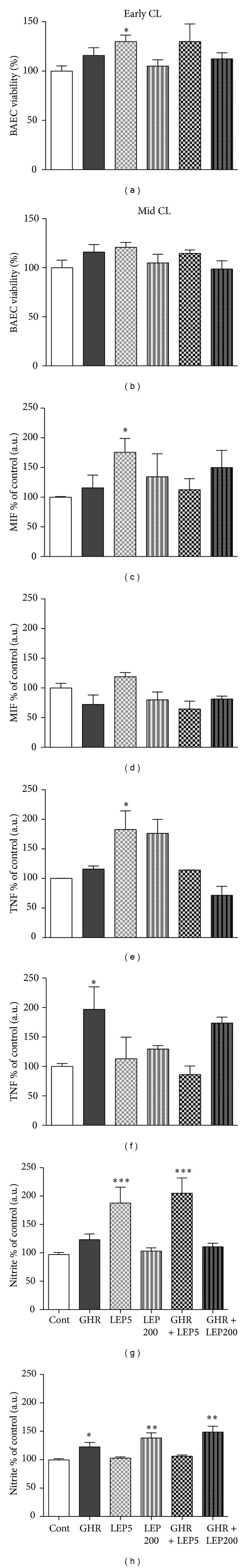
Early CL and mid-CL explants* in vitro* angiogenic activity, assessed after bovine aortic endothelial cell (BAEC) viability measurement ((a) and (b)) and secretion of migration inhibitory factor (MIF, (c) and (d)); tumor necrosis factor *α* (TNF, (e) and (f)); and nitrite ((g) and (h)), after 24 h treatment with no exogenous factor = Control or with GHR (50 ng/mL), LEP (5 ng/mL), LEP (200 ng/mL), LEP (5 ng/mL)+GHR (50 ng/mL), or LEP (200 ng/mL)+GHR (50 ng/mL). Bars represent mean ± SEM. Different asterisks indicate significant differences (*P < 0.05; **P < 0.01; ***P < 0.001). Correspondent values of control for hormone production mean ± SEM: TNF (0.24 ± 0.009 ng/40 mg); MIF (27.47 ± 0.75 ng/mg); and nitrite (0.18 ± 0.037 M/mg).

Viability of BAEC after culture with luteal tissue explant conditioned media presented divergent results, suggesting the ability of LEP and GHR to modulate angiogenesis. Conditioned media from early CL treated with LEP 5 ng/mL increased BAEC viability (*P* < 0.05, Figure 5(a)). Accordantly, MIF concentration was increased in culture medium from early CL treated with LEP 5 ng/mL (*P* < 0.05, Figure 5(c)). Concerning TNF production, it was increased in early CL by LEP 5 ng/mL (*P* < 0.05, Figure 5(e)) and in mid-CL by GHR (*P* < 0.05, Figure 5(f)). The nitrite levels raised with LEP 5 ng/mL and GHR + LEP 5 ng/mL in early CL explants (*P* < 0.001, Figure 5(g)) and with GHR (*P* < 0.05, Figure 5(h)), LEP 200 ng/mL (*P* < 0.01, Figure 5(h)), and GHR+LEP 200 ng/mL in mid-CL (*P* < 0.01, Figure 5(h)).

## 4. Discussion

The need to uncover the role of metabolic factors in reproductive function mainly depends on the tight link between body weight and subsequently energy balance and fertility. For instance, the first adipokine to be described, LEP, has been recurrently associated with food intake regulation, body weight, and energy balance [35]; nonetheless, LEP was also shown to regulate ovarian function, in particular the CL in human [36], porcine [26], and bovine [27]. Moreover, the local auto- and paracrine role of factors such as LEP and GHR in luteal function modulation is not fully understood. In the present study, the physiologic role of LEP and GHR on equine luteal function regulation was demonstrated. Besides characterizing immunolocalization and changes in expression level of LEP, LEPR, GHR, and Ghr-R1A in mare CL throughout the luteal phase, the ligands LEP and GHR were shown to differently modulate luteal secretory activity (P_4_ and PG_s_), TNF, MIF, and nitrite production and angiogenic function. In general, the present results uncover the biphasic role of LEP in luteal secretory activity, as well as the opposing role of LEP and GHR, suggesting that LEP might be associated with CL establishment and GHR with luteolysis.

Immunolocalization of both LEP and LEPR in steroidogenic and endothelial luteal cells invites us to consider the putative effect of LEP system on local steroidogenesis and angiogenesis modulation. In rat ovary [37] and buffalo CL [38] the colocalization of this system in both cell types evidenced the simultaneous action of LEP signaling in steroidogenesis and angiogenesis. This may hold true also for the equine CL. Additionally, the stage-dependent regulation of mRNA and protein production throughout the luteal phase suggests a physiologic action during CL function. Indeed, LEP action in equine CL seems to be controlled by LEPR expression, which was associated with CL growth and establishment. Both mRNA and protein levels of LEP were stable throughout the luteal phase, while those from LEPR decreased from mid- to late CL. Similar findings were obtained in buffalo CL, except that both LEP and LEPR expressions decreased from mid- to late CL [38]. Likewise, in bovine CL these factors presented higher expression level during CL establishment [27]. Leptin expression level in the ovary changes during human menstrual cycle and it was correlated with plasma P_4_, reaching its peak during the luteal phase [39]. In cow [40] and pig CL [26] leptin and its receptor expression increase in association with luteinization and decline alongside the luteal regression.

The other studied factors, GHR and Ghr-R1A, are also expressed in equine luteal steroidogenic and endothelial cells. In general, GHR and its receptor mRNA and protein were observed in young and mature CL in rat, human, and sheep [17–19]. A single earlier study on sheep reproductive tract reported the immunolocalization of GHR in luteal endothelial cells and investigated the role of this factor in luteal angiogenesis [19]. Regarding gene expression analysis, while no changes in GHR mRNA level were found between early CL and mid-CL, protein expression progressively increased from early CL to mid-CL. Additionally, Ghr-R1A mRNA and protein expression increased in the late CL. The raise of Ghr-R1A in the late CL and the highest expression of both ligand and receptor at this stage of the cycle suggest an active participation of GHR signaling pathway during equine CL regression. In a very recent report, an extensive characterization of GHR and Ghr-R1A expression in the bovine reproductive tract showed their expression in bovine CL [41], but no association between GHR signaling and luteolysis was done. Nonetheless, Rak-Mardyła et al. [42] showed that GHR expression increases in the latest stage of pig luteal phase.

In the second part of the study, the role of GHR and LEP in secretory activity and angiogenic function was addressed in both early CL and mid-CL. In fact, in these two stages of the luteal phase it is possible to study the main regulatory mechanisms of CL function. On the one hand, in early CL all biological pathways mediating CL growth are activated; on the other hand, in mid-CL the enzymatic apparatus mediating luteolysis is already responsive to luteolytic stimulus, allowing for the study of CL regression [4, 5, 43].

The analysis of P_4_, PGE_2_, and PGF_2*α*_ secretion interestingly revealed that LEP and GHR present opposing effects. The association of both factors reverted the effect seen when treatments were done individually. This was true for P_4_ and PGE_2_ secretion by early CL and P_4_ production by mid-CL, where LEP supportive effect was reverted by the addition of GHR to the treatment. Apparently, the luteotrophic effect of LEP might be reverted by the antiluteotrophic and/or luteolytic role of GHR. Moreover, a dose-dependent effect of LEP on equine early CL secretion of P_4_ and PGE_2_ is worth noting. This biphasic effect of LEP was previously reported in porcine ovary [26]. In the mentioned study,* in vitro* leptin treatment with low dose (10 ng/mL) increased P_4_ accumulation by luteinized granulosa cells, whereas the high dose (1000 ng/mL) had an inhibitory effect. In a more recent study in rat ovary, the* in vivo* administration of different LEP doses activated distinctive steroidogenic enzymes, with particular emphasis for 3-beta-hydroxysteroid dehydrogenase (3*β* HSD) modulation, in a dose-dependent manner [44]. In our model, we could also see that early CL LEP treatment in a lower dose (5 ng/mL) increased P_4_ secretion, while the higher dose (200 ng/mL) caused no effect. Undeniably, LEP response in equine ovary appears to be dependent on its dose. The previous conclusion holds true for the other studied products, particularly PGE_2_, TNF, MIF, and nitrite quantification in early CL media. Overall, the present findings endow LEP with a broad intervention in CL establishment. Our previous reports clearly evidenced the supportive actions TNF and NO on P_4_ output during CL establishment [43]. Clearly, LEP not only plays a straightforward effect on P_4_ output, but also promotes the secretion of other factors that may support CL growth, such as PGE_2_, TNF, and NO [3, 5, 33, 43].

Another critical event for CL establishment as an endocrine organ is the vascular proliferation [30, 33]. To the best of our knowledge, this is the first evidence of LEP action on luteal angiogenesis. Indeed, LEP increased angiogenic activity and promoted the secretion of TNF, NO, and MIF. As previously demonstrated by our group, TNF and NO are known as proangiogenic factors during equine CL growth [3, 30]. Moreover, TNF itself stimulates NO production, via endothelial NO synthase expression in early CL [43]. Also, TNF promotes angiogenic activity and vascular endothelial growth factor-A expression [33]. Thus, the crosstalk between LEP, TNF, and NO seems to be determinant for vessels proliferation in equine CL growth. It should be noted that MIF quantification as a vasculogenesis marker was based on its well-characterized* in vivo* and* ex vivo* proangiogenic function [45]. Additionally, MIF involvement in highly dynamic vasoproliferative events, such as tumor-associated angiogenesis [46], qualifies this immune regulator as a conventional marker for luteal angiogenesis. Definitely, the celerity of luteal angiogenesis was previously compared with tumor angiogenesis [47]. Besides being exclusively considered as an angiogenic marker in the present study, MIF involvement in bovine CL growth was previously shown [48]. Yet, further studies are needed to better characterize the role of MIF in equine CL function.

Another important observation to be discussed is the involvement of GHR in P_4_ and PGF_2*α*_ secretion. The inhibition of P_4_ secretion from early CL and mid-CL explants indicates that GHR signaling pathway may target P_4_ synthetic enzymes during luteolysis promotion. Indeed, GHR decreased 3*β* HSD expression in porcine CL [42]. Similar findings were reported* in vitro* in human luteal cells [49], where GHR decreased human chorionic gonadotrophin triggered P_4_ secretion and increased PGF_2*α*_. In the present study PGF_2*α*_ was also increased by GHR in mid-CL. Also, GHR involvement in luteolysis is supported by the fact that TNF and nitrite secretions were also increased by this factor. As previously reported in equine [43] and bovine CL [50], nitrite and TNF were both shown to be involved in luteolysis, by directly increasing PGF_2*α*_ or interacting with other cytokines, promoting structural luteolysis [4]. Thus, GHR may be integrated in the local auto- and paracrine set of interactions responsible for the amplification of luteolytic signal in equine CL. Noteworthy, the association of LEP with GHR treatment reverted its luteolytic effect in mid-CL.

Regarding NO, the present response of nitrite production to LEP 200 ng/mL treatment is hard to justify under physiologic conditions. As previously demonstrated, LEP in high doses induces oxidative stress of human endothelial cells* in vitro* [51], and in hyperleptinemic states such as obesity LEP has been associated with increased oxidative stress through different mechanisms [52]. Thus, the present treatment with LEP 200 ng/mL may initiate an oxidative response, which opposes the luteotrophic dose of 5 ng/mL. Nevertheless, further studies should be conducted in the mare to better understand the response mechanisms to euleptinemia and hyperleptinemia conditions.

In conclusion, this study demonstrates the presence of* LEP* and* GHR* systems in the equine CL. On one hand, the luteosupportive role of* LEP* is evidenced by P_4_, PGE2, and TNF secretion and angiogenesis promotion (through angiogenic activity, MIF, TNF, and NO) in early CL; on the other hand, the luteolytic role of* GHR* is mainly mediated by the stimulatory effect on PGF_2*α*_, NO, and TNF in mid-CL. Finally, a dose-dependent luteotrophic effect of* LEP* was demonstrated, as well as the opposing roles of* LEP* and* GHR* in equine CL regulation.

## Figures and Tables

**Table 1:** Specific primer sequences used for quantitative real-time PCR.

Gene	Accession number	Sequence 5′-3′	Length (base pairs)
*LEP *	NM_001163980.1	For: CACGCAGTCAGTCTCCTCCA	101
		Rev: TTGCCAATGTCTGGTCCATC	
*LEPR *	XM_005610519.1	For: CCCACTTCATCGCCAAAAGA	179
		Rev: CCCATTTGATCACAGCCACA	
*GHR *	XM_001491134.4	For: GTTCAACGCCCCCTTTGAT	101
		Rev: CCTCCCAGAGGATGTCCTGA	
*Ghr-R1A *	XM_001494000.1	For: TCATCAGCAGGAAGCTGTGG	178
		Rev: CCAGGCTCAAAGGATTTGGA	
*B2MG *	X69083	For: CGGGCTACTCTCCCTGACTG	92
		Rev: TTGGCTTTCCATTCTCTGCTG	
